# Analyzing Satellite Imagery to Target Tuberculosis Control Interventions in Densely Urbanized Areas of Kigali, Rwanda: Cross-Sectional Pilot Study

**DOI:** 10.2196/68355

**Published:** 2025-04-24

**Authors:** Mauro Faccin, Caspar Geenen, Michiel Happaerts, Sien Ombelet, Patrick Migambi, Emmanuel André

**Affiliations:** 1 Department of Medical and Surgical Sciences, University of Bologna, Via Massarenti 9, Bologna, 40126, Italy, 39 051 2143578; 2 Istituto Nazionale di Fisica Nucleare, Sezione di Bologna, Bologna, Italy; 3 Department of Microbiology, Immunology and Transplantation, Laboratory of Clinical Microbiology, KU Leuven, Leuven, Belgium; 4 Department of General Internal Medicine, University Hospital Leuven, Leuven, Belgium; 5 Department of Laboratory Medicine, University Hospital Leuven, Leuven, Belgium; 6 Tuberculosis and Other Respiratory Disease Division, Department of HIV/AIDS Diseases Prevention and Control, Rwanda Biomedical Center, Kigali, Rwanda

**Keywords:** tuberculosis, Rwanda, satellite image, TB, PCR testing, PCR, questionnaire, satellite, active case-finding, diagnostic, urban, Africa, TB screening, ACF, polymerase chain reaction

## Abstract

**Background:**

Early diagnosis and treatment initiation for tuberculosis (TB) not only improve individual patient outcomes but also reduce circulation within communities. Active case-finding (ACF), a cornerstone of TB control programs, aims to achieve this by targeting symptom screening and laboratory testing for individuals at high risk of infection. However, its efficiency is dependent on the ability to accurately identify such high-risk individuals and communities. The socioeconomic determinants of TB include difficulties in accessing health care and high within-household contact rates. These two determinants are common in the poorest neighborhoods of many sub-Saharan cities, where household crowding and lack of health-care access often coincide with malnutrition and HIV infection, further contributing to the TB burden.

**Objective:**

In this study, we propose a new approach to enhance the efficacy of ACF with focused interventions that target subpopulations at high risk. In particular, we focus on densely inhabited urban areas, where the proximity of individuals represents a proxy for poorer neighborhoods with enhanced contact rates.

**Methods:**

To this end, we used satellite imagery of the city of Kigali, Rwanda, and computer-vision algorithms to identify areas with a high density of small residential buildings. We subsequently screened 10,423 people living in these areas for TB exposure and symptoms and referred patients with a higher risk score for polymerase chain reaction testing.

**Results:**

We found autocorrelation in questionnaire scores for adjacent areas up to 782 meters. We removed the effects of this autocorrelation by aggregating the results based on H3 hexagons with a long diagonal of 1062 meters. Out of 324 people with high questionnaire scores, 202 underwent polymerase chain reaction testing, and 9 people had positive test results. We observed a weak but statistically significant correlation (*r=*0.28; *P*=.04) between the mean questionnaire score and the mean urban density of each hexagonal area.

**Conclusions:**

Nine previously undiagnosed individuals had positive test results through this screening program. This limited number may be due to low TB incidence in Kigali, Rwanda, during the study period. However, our results suggest that analyzing satellite imagery may allow the identification of urban areas where inhabitants are at higher risk of TB. These findings could be used to efficiently guide targeted ACF interventions.

## Introduction

Tuberculosis (TB) is one of the major global health challenges that could be managed through prompt diagnosis and treatment initiation. An estimated 10.6 million people became ill, and 1.6 million people died from TB in 2021 [1]. Despite international efforts, the burden of disease remains uncontrolled in many of the most fragile communities around the world. As poverty-affected communities experience major difficulties in accessing quality medical care, screening for TB in these populations cannot rely solely on health care–centered diagnosis. Considering that disease notification by routine diagnostic activities imperfectly reflects the burden of disease in such populations, alternative case detection approaches are needed.

To this end, community-based active case-finding (ACF) can be an effective approach if targeted at high-risk subgroups or implemented with high coverage and intensity [1-5]. However, the yield of ACF is difficult to predict, and cost-efficiency can be questioned when a large number of people need to be screened to identify a few infected individuals [6]. In practice, large-scale screening at the community level is usually done using a simple triage tool, followed by selective laboratory testing. These triage tools typically include AI-assisted chest X-ray radiography or symptom screening [7,8]. However, even these triage tests can be difficult to scale up and are inefficient if deployed in low-risk communities. In a previous study [8], we showed that the burden of TB could be accurately predicted in rural environments of the Democratic Republic of the Congo by integrating historical disease notification, distance of villages to the nearest health care center, and proximity to mining activities.

Africa accounted for a quarter of all new TB cases worldwide in 2022 and has been at the center of many efforts to eradicate TB [1]. Since 1990, the continent has seen rapid urbanization, with 0.5 billion more people now living in urban areas [9]. While population density is generally associated with easier geographical access to health services, rapid urbanization is also intrinsically associated with socioeconomic disparities, including the development of crowded townships [10]. Due to these disparities and the absence of specific interventions targeting the specific health needs of poverty-affected neighborhoods, TB control in urban areas may be delayed as pockets of TB are maintained by the cycle of disease and poverty.

Rwanda has moved into a pre-eradication phase regarding TB, with an estimated incidence of 56 patients per 100,000 population in 2021, a decline of more than 40% over the past two decades [1]. Nevertheless, the city of Kigali has a higher incidence of reported TB (108 per 100,000 inhabitants yearly) compared to the rest of the country.

In previous studies, metrics derived from satellite images have correlated with the prevalence of both noncommunicable and communicable diseases, particularly when affected by climate [11,12].

The aim of this study was to assess the feasibility of guiding targeted ACF interventions in an urban environment (ie, Kigali, Rwanda) using satellite images [13]. We hypothesized that those urban areas with a high density of small residential buildings—as a proxy for poverty and crowded households—are associated with a high risk of undiagnosed TB [14]. We constructed an urban density index (UDI) to highlight such areas, dispersed throughout the city, and investigate the correlation with TB screening results.

## Methods

### Study Design

In this cross-sectional pilot study, we combined analysis of satellite images with results of ACF for TB in July 2021 in Kigali, Rwanda.

### Urban Density Index Based on Satellite Images

**Figure 1.**
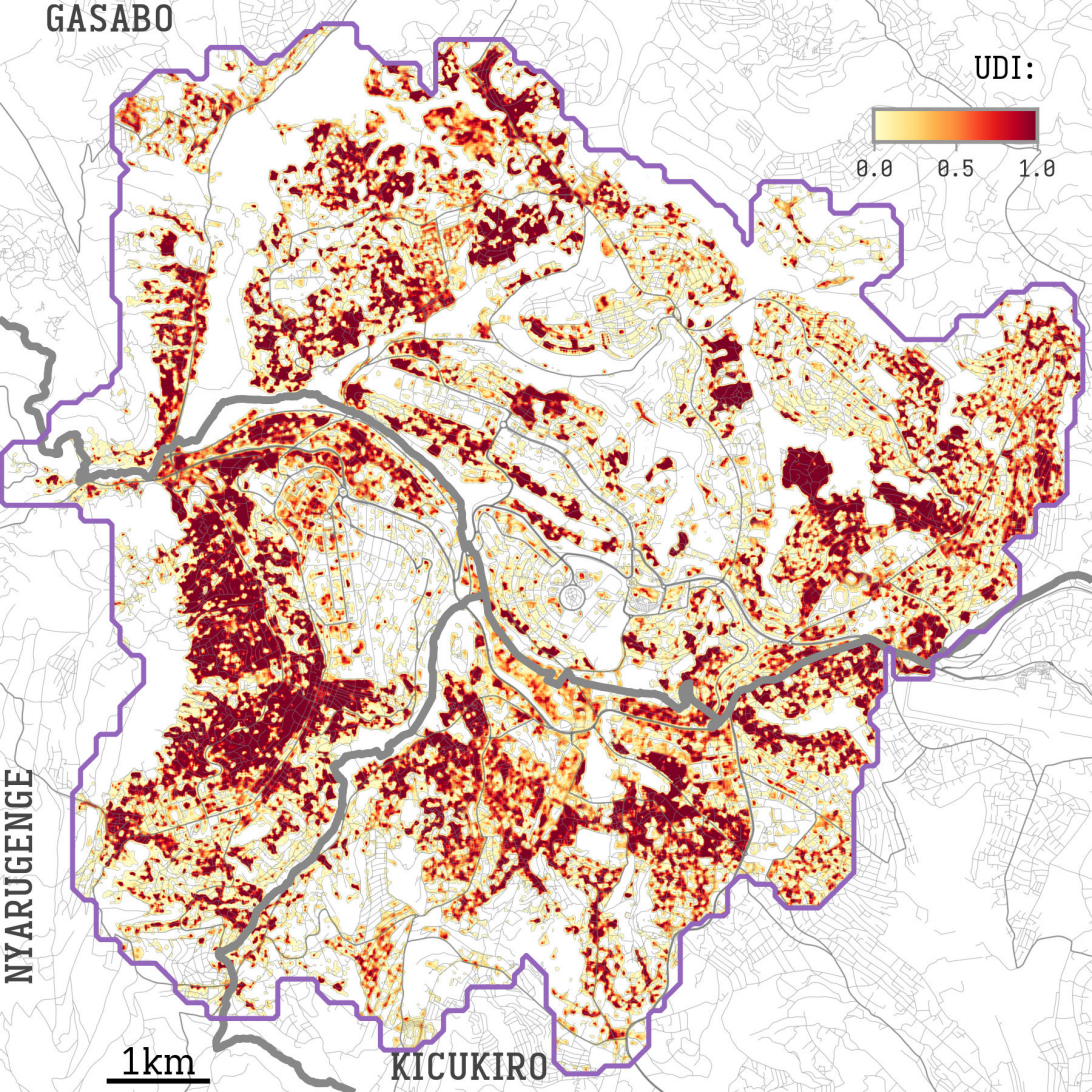
Urban density index (UDI) overlaid on a map of Kigali. A white-to-red color scale indicates the UDI. High UDI values correspond to a high density of small buildings and a low density of green spaces. A purple line indicates the edge of the city, as defined by an estimated population density over 6000 per square kilometer.

The urban area to be analyzed was delineated based on the population density provided by WorldPop [15], selecting the cells that are estimated to have more than 8000 inhabitants per square kilometer (see the purple boundary in Figure 1). This area was extended to surrounding cells by filling holes and dilating to neighboring cells within 5 steps (approximately 500m).

We used a two-step computer-vision approach applied to the satellite imagery to calculate a UDI. Satellite images of the urban area of interest were retrieved from ArcGIS (source: Esri, Maxar, Earthstar Geographics, and the GIS User Community) at zoom level 17 (approximately 1 meter per pixel). All tiles intersecting the area of interest were joined in a large image.

The first step detects object contours from the prepared image with the aim to highlight zones with a high density of small houses or shelters. To perform such detection, we used the contour-detection Canny algorithm. The latter is a multistage algorithm that detects edges of an image by computation of the intensity gradient. Cells with high values of intensity gradient were selected to form an edge image. After blurring (blurring radius of approximately 50m), areas with a higher density of edges had higher values.

In the second step, we selected and discarded all green areas in the satellite images. As a result, in areas such as open fields and gardens, as well as within wealthier neighborhoods—where one can assume larger houses surrounded by gardens or public green—the index was set to zero. The green areas were detected applying a hue, saturation and value color filter to the satellite images, selecting all pixels with hue in the range of 15‐120 and value below 120.

The result was a spatial UDI map with a resolution of approximately 1m per pixel, with the UDI ranging from 0 to 1.

### Active Case Finding

Based on the selection of high-risk areas by the UDI, seven sectors (ie, Nyakabanda, Kimisagara, Rwezamenyo, Nyaregenge, Kimironko, Remera, and Niboye) were visited by 10 community health workers (CHW) during 21 days in July 2021 to perform door-to-door screening surveys. All persons residing at the visited residential locations were eligible to participate. The number of participants was determined by how many persons the CHWs could visit during this time frame.

In total, 10,423 persons were screened using MediScout (Savics, Belgium), a digitally supported questionnaire adapted from a previous study [8]. The screening and selection process was conducted by the local National TB Program after they were given access to the UDI map.

The questionnaire (see Table S1 in Multimedia Appendix 1 for the list of questions) was composed of three sets of questions evaluating the symptoms, environment, and health history of the individual. This questionnaire was composed of a set of seven questions formulated in Kinyarwanda and was administered by trained CHWs.

The weighted responses assigned to each screened individual provided a personal total score between 0 and 18 (Qscore). When the Qscore ≥4, the CHW suggested that the individual provide a sputum sample, which was analyzed using an Xpert MTB/Rif Ultra polymerase chain reaction (PCR) assay (Cepheid) [16]. The samples collected were transferred to the laboratory, and tests were performed within 24 hours after collection.

The answers to the questionnaire, together with the exact location where the survey took place, were collected using the MediScout app (Savics, version 1.0).

### Disaggregation Length-Scale

Analysis at the level of the three city districts did not reveal major disparities in average Qscores. We then investigated whether UDI correlated with tuberculosis risk within a more restricted spatial range. To determine the optimal aggregation level, we investigated the spatial autocorrelation of risk scores. To this end, we used the global Moran’s I [17,18], a statistical measure of autocorrelation of spatial data. We confirmed their results through a variogram analysis [19] (see Figure S1 in Multimedia Appendix 1). A variogram is a tool from geostatistical modeling that quantitatively assesses semivariance over a variable lag distance. This tool assesses the spatial autocorrelation of the data and estimates the distance at which those correlations vanish. We found that the estimated maximum distance at which nearby risk scores were correlated was approximately 782 meters.

**Figure 2.**
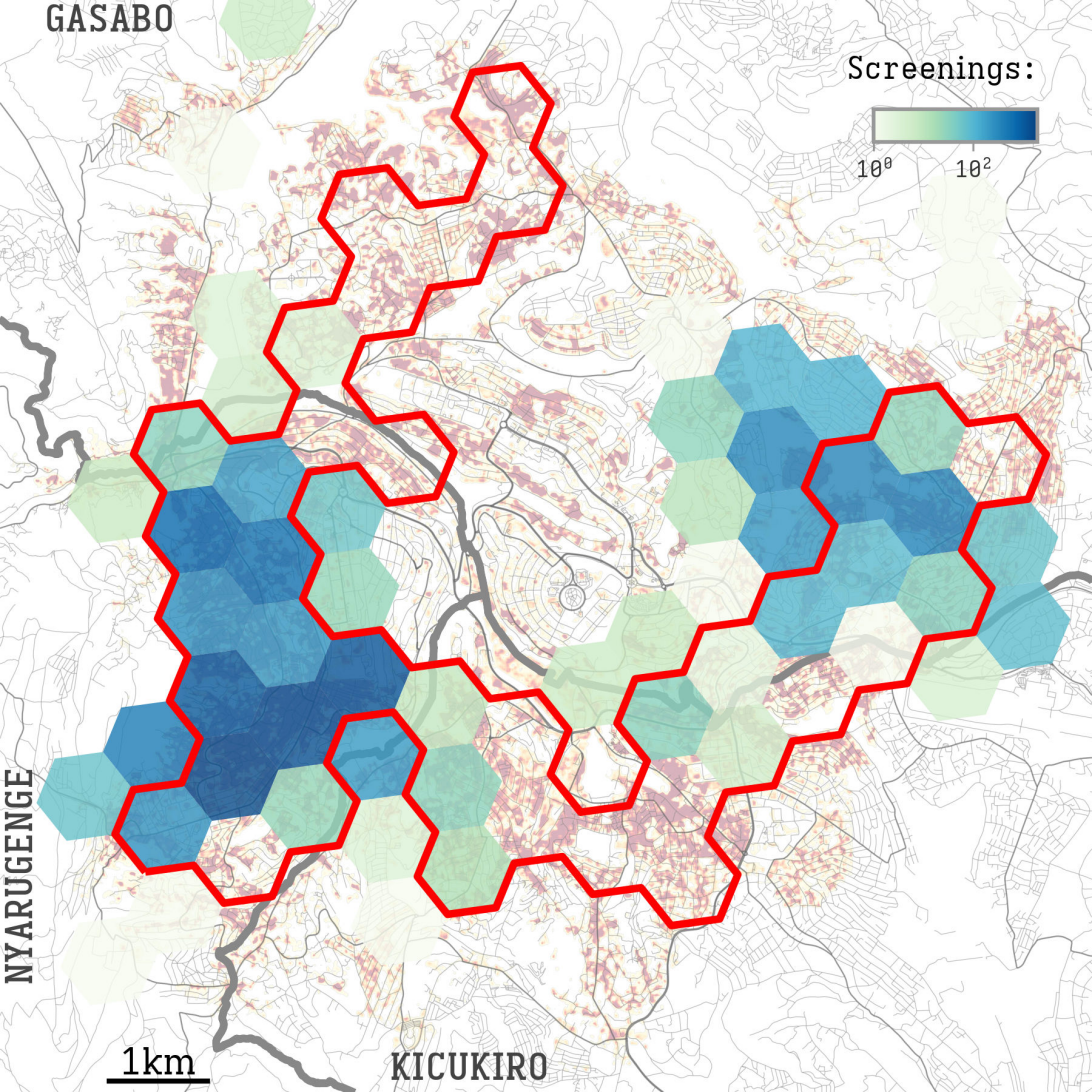
Distribution of screenings in Kigali neighborhoods. A color scale shows the number of screening interviews performed per hexagonal area, overlaid on the urban density index (UDI) map of Figure 1. Screenings were broadly targeted to high-UDI neighborhoods, after visual inspection of Figure 1, and then aggregated in H3 hexagons. The area delineated in red shows hexagons with a high UDI (>0.353).

Therefore, we performed a segmentation of the city area based on H3 hexagons [20] with a long diagonal of 1062 meters, the smallest segmentation level that removed the observed autocorrelation. We assumed that the risk inside each hexagon was homogeneous, despite the heterogeneity of the enclosed neighborhoods. These hexagons had a mean estimated population of 8385 (SD 4688) inhabitants. A hexagon was labeled as “high UDI” if it had a mean UDI >0.353, the 75th percentile of the city surface UDI shown in Figure 1. Figure 2 overlays these high UDI hexagons with the total number of screenings performed in each hexagon. See Figure S2 in Multimedia Appendix 1 for deeper analysis. The resulting hexagons with the aggregated screenings and UDI are included in Multimedia Appendix 2.

### Statistical Analysis

Proportions were reported using binomial test confidence intervals and compared with the Fisher exact test. The aggregation of the dataset on the hexagonal grid allowed us to treat the data points as statistically independent. In regression analysis, we used the t statistic to compute the *P* values of the slope.

Persons who completed the questionnaire but did not complete PCR testing were considered lost to follow-up, and were not included in further analysis.

### Ethical Considerations

The study protocol was approved by the Rwanda National Ethical Committee (Institutional Review Board 00001497 of IORG0001100). Informed consent was electronically obtained from all screened participants through the MediScout app. No personal identifiers were collected except for the location of the screening, sex, and age; these data were securely stored in a password-protected database. No compensation was provided to the participants.

## Results

### Visual Validation of the Urban Density Index

**Figure 3.**
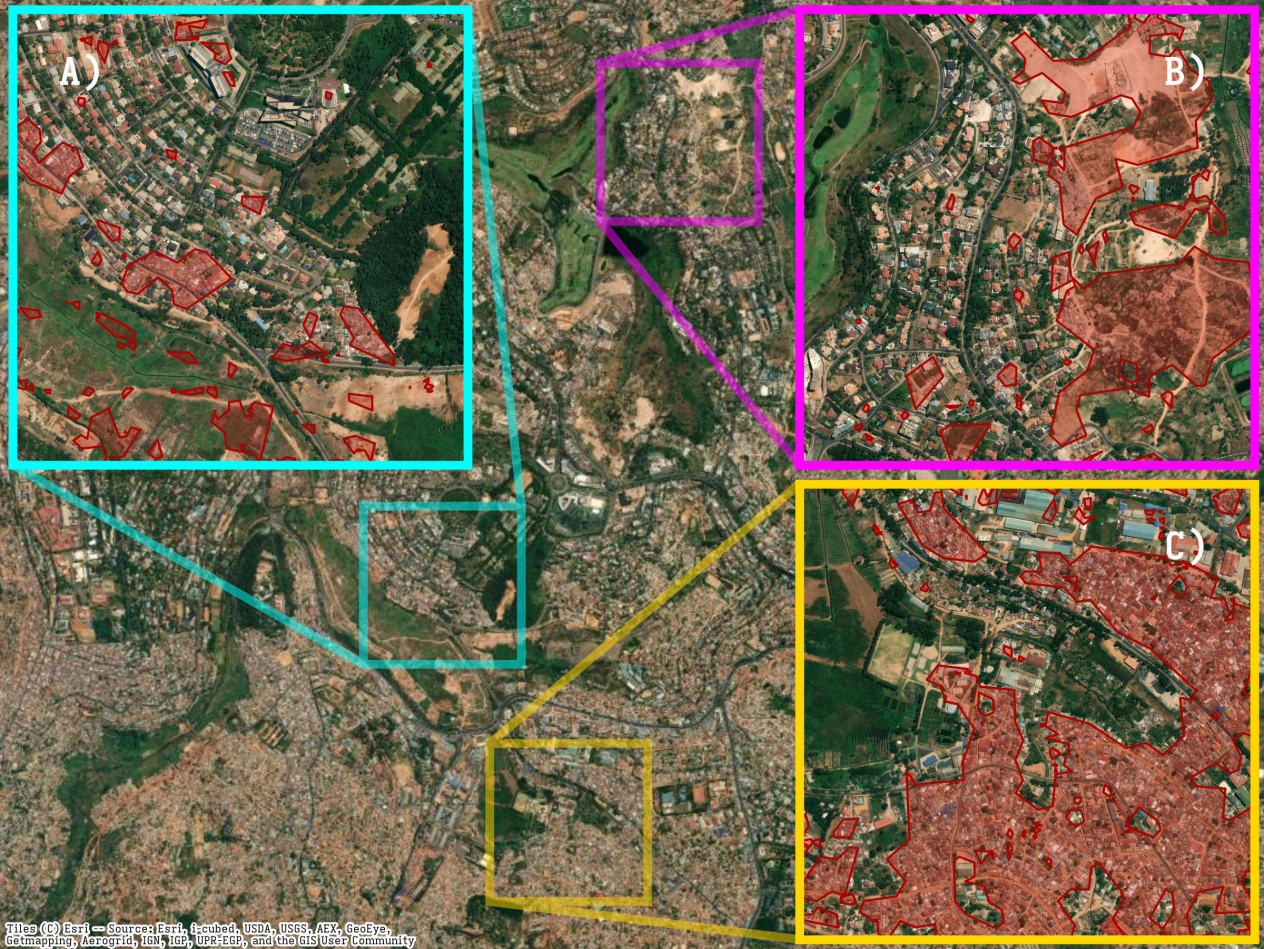
Overlay of the UDI (red highlights indicate UDI >0.5) onto satellite images of Kigali, providing a visual indication of the algorithm’s ability to automatically identify areas with a high density of small residential buildings and few green spaces. Inlays highlight (A) areas with small clusters of dense shelters within a wealthy neighborhood; (B) a wealthy and poor neighborhood side by side; (C) dense neighborhoods surrounding green areas.

First, we analyzed satellite images to produce a UDI map of Kigali for each square meter (Figure 1). A visual inspection of selected areas (Figure 3) confirmed a clear overlap between high UDI and the density of small buildings and scarcity of green spaces. This figure highlights three areas of Kigali with different building density distributions: small but dense clusters of buildings within healthier neighborhoods (Figure 3A); adjacent neighborhoods with differing density of buildings (Figure 3B); and dense neighborhoods surrounding green areas (Figure 3C). In all cases, the UDI identified areas with a high density of buildings.

Locations with UDI >0.5 accounted for 29% of the total surface of the city. Table 1 provides an overview of notified TB cases and UDI in the different sectors of Kigali. For each sector, the table shows the population estimate reported by the Rwandan National Institute of Statistics [21], the total number of confirmed cases reported by the Ministry of Health [22], and the resulting estimate of incidence (per year per 100,000 inhabitants). The surface area with UDI greater than 0.5 is also reported as a summary statistic.

**Table 1.** Urban density index and incidence of confirmed cases of tuberculosis by sector of Kigali.

Sector	Population	TB^a^ cases (2017)	Incidence rate	Surface UDI^b^ >0.5 (%)
Gasabo	879,505	570	65	27
Kicukiro	491,731	533	108	34
Nyarugenge	374,319	790	211	39
Kigali (aggregated)	1,745,555	1893	108	31

a TB: tuberculosis.

b UDI: urban density index.

### Targeting of Active Case Finding Interventions Based on Urban Density Index

In total, the CHWs screened 10,423 individuals (Table 2, Figure S3 and S4 in Multimedia Appendix 1). In Nyarugenge district, which had the highest TB notification rate, 3.3% (n=270) of screened individuals had a Qscore ≥4, and this proportion was similar in the Gasabo district, which had the lowest notified TB incidence: 2.6% (n=51; *P*=.13). Mean Qscores in Nyarugenge and Gasabo among screened individuals were also similar (0.64 and 0.61, respectively; *P*=.34). Age was positively associated with Qscore (*P*<.001).

**Table 2.** Tuberculosis screening results aggregated by administrative district, age, and sex.

Variables	Screenings (N=10,423)	Screenings Q score ≥4	Molecular tests (n=202)	Positive tests (n=9)	Mean Qscore
Sex					
Female	5222	138	77	2	0.60
Male	5197	186	125	7	0.67
Missing‍	4	0	0	0	0.50
Age (years)					
1‐10	435	7	4	0	0.34
11‐20	1539	32	17	0	0.43
21‐30	3098	62	40	2	0.54
31‐40	2619	91	57	3	0.69
41‐50	1483	75	47	4	0.82
51‐60	672	31	18	0	0.84
61‐70	336	16	11	0	0.94
71+	165	9	8	0	1.04
‍Missing	76	1	0	0	0.76
Districts					
Gasabo	1988	51	30	2	0.61
Kicukiro	129	3	3	0	0.53
Nyarugenge	8306	270	169	7	0.64
Kigali (total)	10,423	324	202	9	0.63

### Correlation Between Tuberculosis Risk and Urban Density Index

**Figure 4.**
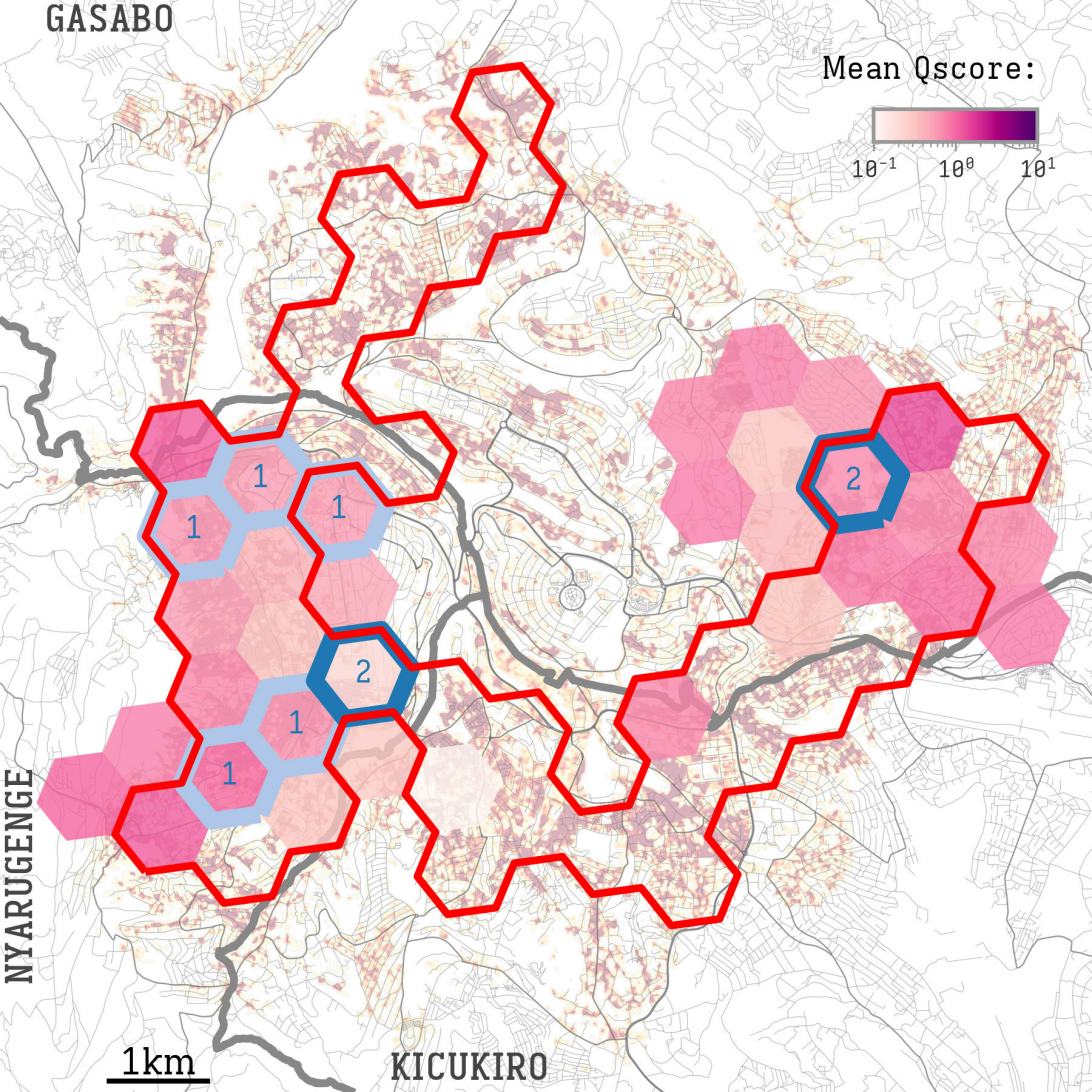
Mean Qscore results. A color scale shows the mean Qscore in each hexagonal cell, overlaid on the UDI map in Figure 1. Only cells with at least 10 screenings are displayed. Tuberculosis cases were found in the cells highlighted in blue, labeled with the number of cases detected. The area delineated in red shows hexagons with a high urban density index (>0.353).

Of all screenings (N=10,423), 8585 (82.4%) were performed in high UDI hexagon cells, and 1838 (17.6%) in low UDI cells (Figure 2). Among the screened individuals, 324 (3.1%) were considered at risk for TB based on the questionnaire-derived Qscore ≥4: 254 (3.0% of screenings) in high UDI cells and 70 (3.8% of screenings) in low UDI cells (*P*=.06) (Figure 4). Of the 324 at-risk individuals, 202 (62.3%) provided a sputum sample: 166 (82.2%) in high UDI cells and 36 (17.8%) in low UDI cells.

*Mycobacterium tuberculosis complex* was detected in 9 of 202 (4.5%) individuals using the GeneXpert assay on sputum samples. Eight of the newly detected TB cases lived in high UDI areas, corresponding to a prevalence of 93 in 100,000 (95% 47‐175). In low UDI cells, one individual had positive results, indicating a prevalence of newly detected TB of 54 in 100,000 (95% CI 0‐270).

**Figure 5.**
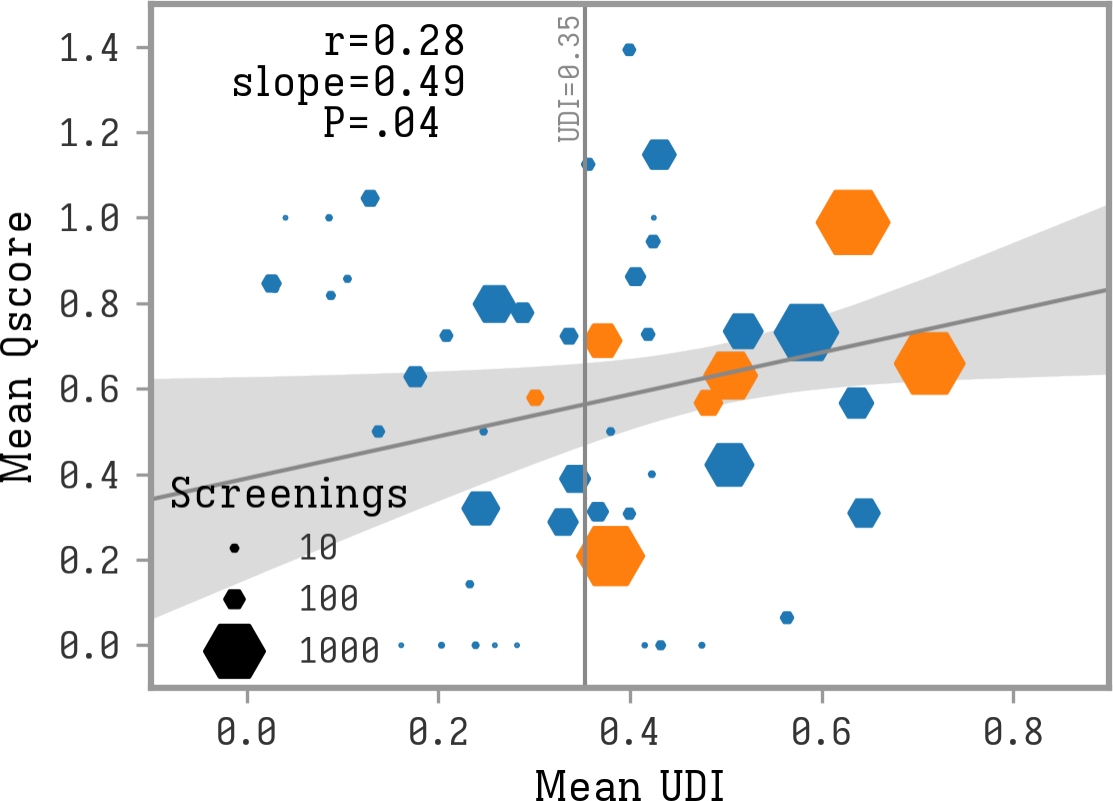
Correlation between mean UDI and mean Qscore. Each hexagon represents a hexagonal geographical area, orange if at least one positive case was found, in blue otherwise. The size of the hexagons indicates the number of screenings performed. A black line and gray confidence band indicate the result of a linear regression analysis weighted by the number of screenings in each area. The threshold to consider an area as high UDI (mean UDI >0.353) is indicated with a vertical line. UDI: urban density index.

We observed a weak (*r=*0.28) but statistically significant correlation between the mean Qscore and mean UDI of each hexagon (*P*=.04) (Figure 5). This suggests that locations with high UDI values, indicating dense areas within the city, tend to have higher Qscores. Given that the Qscore serves as a proxy for TB risk, this correlation implies that areas with higher UDI might experience a greater number of possibly undiagnosed cases.

## Discussion

In this study, we investigated the value of satellite imagery to guide targeted TB control interventions in Kigali, Rwanda. The central finding is that satellite-derived UDIs, which highlight densely populated areas with limited green spaces, could be used to identify communities that share a higher risk of TB.

We assessed this risk using a questionnaire administered to more than 10,000 participants across the city. Individual TB risk was estimated using a personalized score based on a weighted-answer questionnaire previously validated in other contexts [8]. We observed a weak but significant correlation between UDI and TB risk scores. Participants with an elevated risk for TB were offered a PCR test. Of 202 individuals referred for testing, nine cases of TB were confirmed. This corresponds to a prevalence of undetected TB of 86 per 100,000 inhabitants in the general population. Although we observed a correlation between UDI and TB risk scores, this study did not clearly demonstrate the efficient detection of TB cases due to the low number of actual cases detected. However, these findings suggest that satellite imagery could play a valuable role in identifying areas where targeted TB interventions are needed, particularly in resource-constrained urban settings. Our results highlighted that individuals living near each other had a risk for TB, which auto correlated within a radius of around 0.8 km. This information may be useful in planning and delineating targeted screening interventions in areas where new TB cases are identified. In general, only households and close contacts are currently included in ACF interventions. Our results suggest that the perimeter of ACF interventions could be extended to the neighborhood level.

The main limitation of this study was the low number of new TB cases detected, probably due to the relatively low overall prevalence of TB in Kigali during the study. Therefore, the observed correlations of TB risk with UDI were based on questionnaire-based risk scores rather than microbiological confirmation. This study also did not establish a causal link between high-density residential building and prevalence of undetected TB. However, for the purpose of efficiently targeting screening to specific urban areas, a causal link does not need to be shown. It should also be noted that this study took place in a single city, and the results may not be directly generalizable to other settings. Finally, as areas for screening were selected by health care workers based on the UDI map, high UDI areas were overrepresented in the study, resulting in selection bias and reduced statistical power.

We expect that the inclusion of more low UDI areas could have resulted in a stronger observed correlation between UDI and TB risk.

Overall, our study suggests that, with or without satellite image analysis, targeting ACF for TB to small geographic entities (eg, smaller than 1 km) could help leverage geographic disparities to increase the yield of community-based TB control. While local health care systems typically aggregate cases by larger administrative regions, TB infections might exhibit higher incidence within certain smaller areas. Nevertheless, this approach should not substitute individualized contact tracing efforts, considering the population mobility and the contact networks outside the living areas, which are also involved in disease transmission.

## Supplementary material

10.2196/68355 Multimedia Appendix 1 Additional information: detection of local aggregation; hexagonal grid choice; questionnaire replies frequencies; questionnaire original questions.

10.2196/68355 Multimedia Appendix 2 Aggregated data: urban density index and aggregated screening results.

10.2196/68355 Checklist 1 STROBE checklist.

## Abbreviations

ACF: active case-finding
CHW: community health workers
HSV: hue saturation and value
PCR: polymerase chain reaction
TB: tuberculosis
UDI: urban density index
