# Supplementary material for: Analyzing Satellite Imagery to Target Tuberculosis Control Interventions in Densely Urbanized Areas of Kigali, Rwanda: Cross-Sectional Pilot Study
Source: JMIR Public Health Surveill. 2025 Apr 24;11:e68355. doi: 10.2196/68355 (PMC12045519; doi:10.2196/68355)
Supplement: Multimedia Appendix 1 [file publichealth-v11-e68355-s001.pdf]

Supplementary Methods 1. Detection of local aggregation

The Global Moran's I[1] suggest a non-negligible geographic autocorrelation of the questionnaire score (Moran's I=0.10, p-value<0.0001). See[2] for a spatial analysis based on Local Moran's I.

To determine the natural length below which local correlation is expected, we use tools from spatial statistics. The semivariogram[3], for instance, accounts for the local autocorrelation of geographic data. The semivariogram is defined as half of the variance of the difference between any pairs of data points at a given distance (lag). Beyond the lag at which the semivariogram plateaus, called effective range, one can assume that no local correlation is influencing the values. Figure S1 shows the semivariogram of the qscore and highlights that the effective range is of 782 meters (with 95% CI: 602–961).

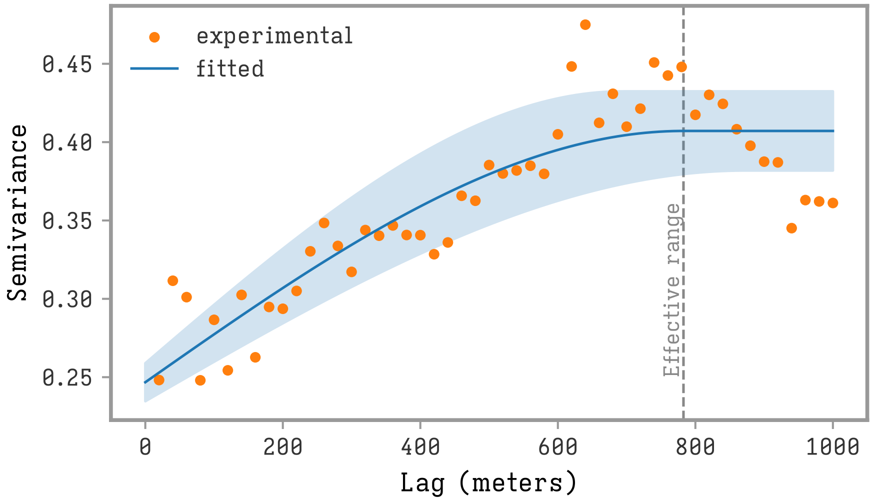

Figure S1: Semi-variogram of the qscore. The lag (r) represents the influence radius below which one can expect local autocorrelations.

Supplementary Methods 2. Hexagonal grid

We aggregated the collected data in a hexagonal lattice (H3 spatial indexing introduced by Uber Technologies Inc.[4]). The 8th level of this lattice representation is a hexagonal grid with an average distance between polygon centres of about 920 meters and provides an aggregation basis that guarantees the absence of cross-cell local correlation.

To validate the above spatial analysis, we computed the Global Moran's I for aggregated data as proposed by[5]. This quantity computes the correlation between the personal score of individuals belonging to neighboring hexagonal cells. See Figure S2. At high granularity (high H3 levels) the correlation is still non-negligible. The latter decreases and eventually reaches zero between H3 levels 7 and 8, supporting the choice of H3 level 8 as an autocorrelation-free aggregation.

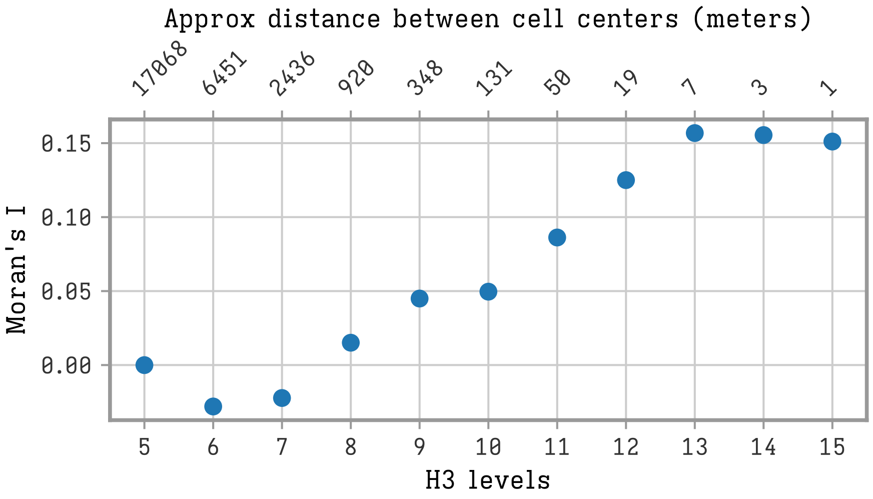

Figure S2: Moran's I as a function of the hexagonal aggregation level. The autocorrelation highlighted by the Moran's I at high H3 levels (high granularity), decreases gradually at lower H3 levels reaching zero between level 7 and 8.

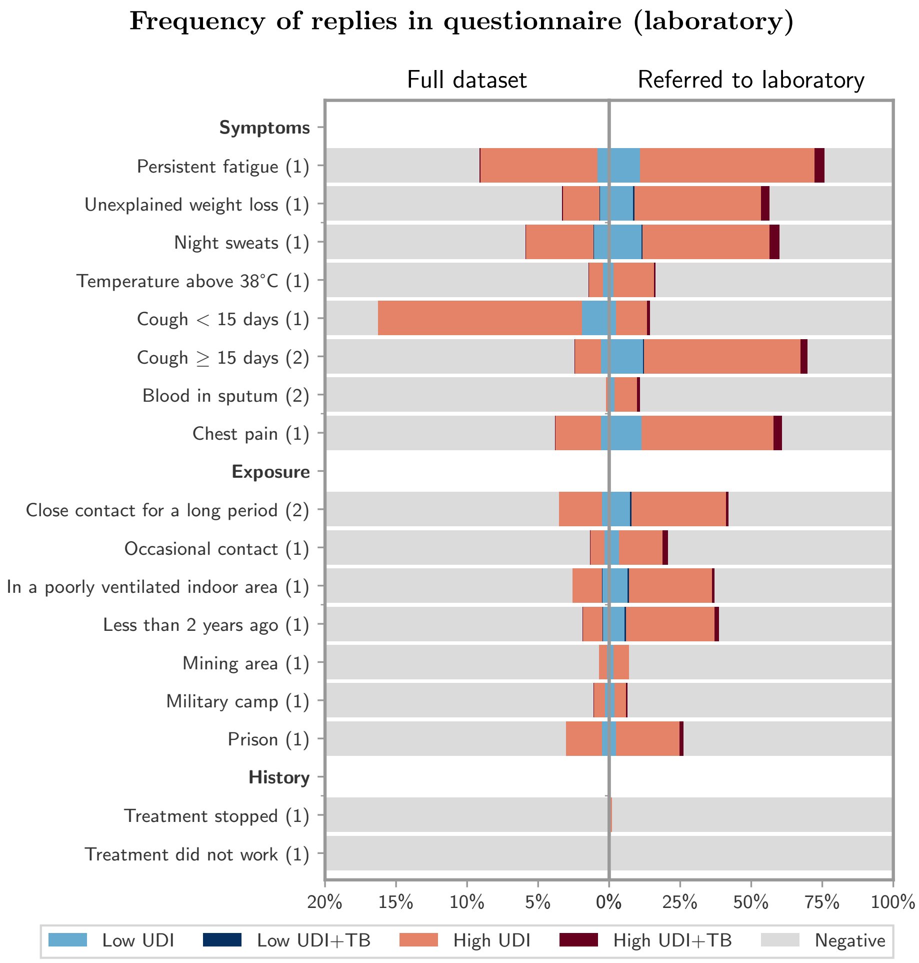

Figure S3: Frequency of replies to the questionnaire (answer weight in parenthesis). Within the full dataset (left) and restricted to the subset of persons which referred to a laboratory for a test.

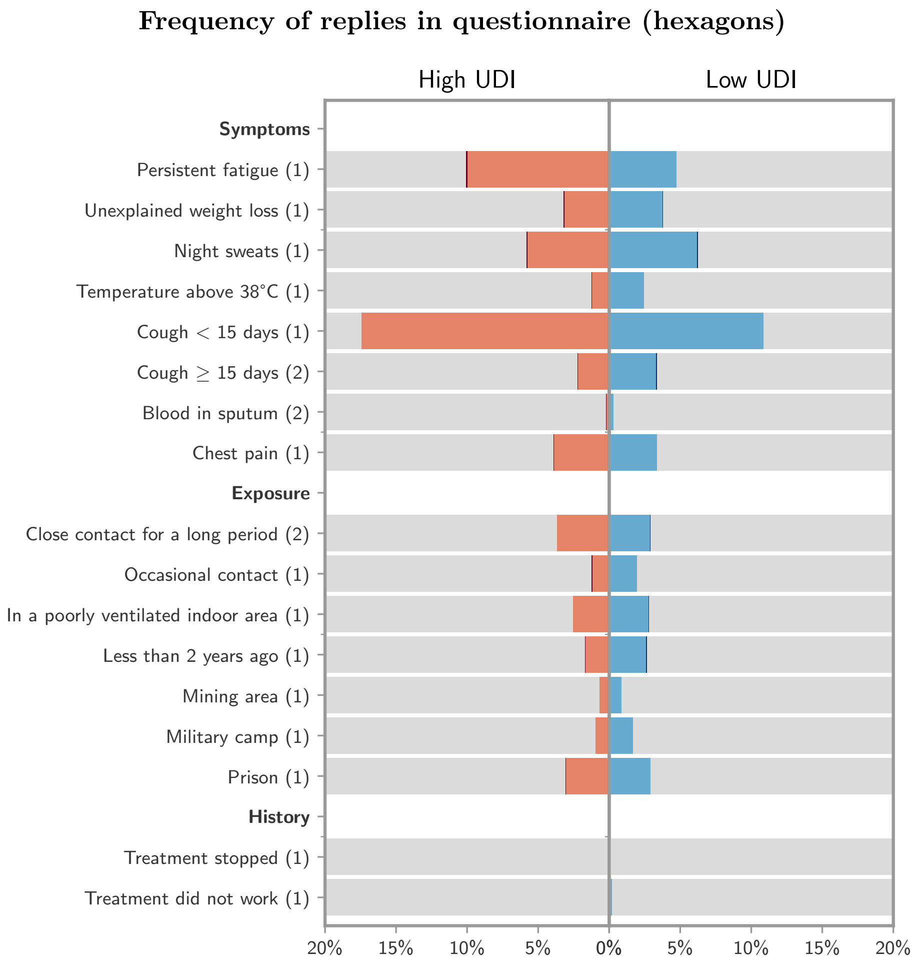

Figure S4: Frequency of symptoms and exposure to TB among screened individuals in high-risk hexagons (left) and low-risk hexagons (right).

Questionnaire

**Do you have any of these general symptoms?**	
Persistent fatigue	1
Unexplained weight loss	1
Night sweats	1
Temperature above 38°C (Measurements taken during the interview)	1
None of the above	0
**Do you have any of these pulmonary symptoms? (More than one answer is allowed)**	
Cough (less than 15 days)	1
Cough (more than 15 days)	2
Blood in sputum	2
Chest pain	1
None of the above	0
**Do you personally know someone who has (or had) tuberculosis? (Only one answer is allowed)**	
No	0
Yes (close contact for a long period of time)	2
Yes (occasional contact)	1
Yes (no direct contact)	0
**If you had direct contact with someone who has (or had) tuberculosis, this contact took place: (Only one answer is allowed)**	
In a poorly ventilated (indoor) area	1
In a well-ventilated place (outdoor)	0
I had no direct contact	0
**If you had direct contact with someone who has (or had) tuberculosis, this took place: (Only one answer is allowed)**	
Less than 2 years ago	1
More than 2 years ago	0
I had no direct contact	0
**Have you ever been treated for tuberculosis? (Only one answer is allowed)**	
No	0
Yes (cured)	0
Yes (treatment in progress - RHZE)	0
Yes (treatment stopped)	1
Yes (treatment did not work)	1
Yes (treatment in progress - MDRTB)	0
**Do you live or have you ever lived in a place considered a priority for TB control? (More than one answer is allowed)**	
Mining area	1
Military camp	1
Prison	1
None of these places	0

Table S1: Questionnaire used in this study with the corresponding weights. The questions and answers are translated into English from the original version in Rwandan.

Supplementary References

1. P. A. P. Moran. Notes on continuous stochastic phenomena. *Biometrika*, 37 (1–2):17–23, 1950. ISSN 1464-3510. doi: 10.1093/biomet/37.1-2.17. URL http://dx.doi.org/10.1093/BIOMET/37.1-2.17.

2. M. Molemans, L. Kayaert, Q. Olislagers, S. Abrahams, N. Berkowitz, E. Mohr-Holland, D. McKelly, R. Wood, F. van Leth, and S. Hermans. Neighbourhood factors and tuberculosis incidence in cape town: A negative binomial regression and spatial analysis. *Tropical Medicine & International Health*, May 2024. ISSN 1365-3156. doi: 10.1111/tmi.14001. URL http://dx.doi.org/10.1111/tmi.14001.

3. Georges Matheron. Principles of geostatistics. *Economic Geology*, 58(8): 1246–1266, December 1963. ISSN 0361-0128. doi: 10.2113/gsecongeo.58.8.1246. URL http://dx.doi.org/10.2113/gsecongeo.58.8.1246.

4. Vojtěch Uher, Petr Gajdoš, Václav Snášel, Yu-Chi Lai, and Michal Radecký. Hierarchical hexagonal clustering and indexing. *Symmetry*, 11(6):731, May 2019. ISSN 2073-8994. doi: 10.3390/sym11060731. URL http://dx.doi.org/10.3390/sym11060731.

5. Yufan Wang, Wangyong Lv, Minjian Wang, Xu Chen, and Yao Li. Application of improved moran's i in the evaluation of urban spatial development. *Spatial Statistics*, 54:100736, April 2023. ISSN 2211-6753. doi: 10.1016/j.spasta.2023.100736. URL http://dx.doi.org/10.1016/j.spasta.2023.100736.
